# The effect of glia-glia interactions on oligodendrocyte precursor cell biology during development and in demyelinating diseases

**DOI:** 10.3389/fncel.2013.00268

**Published:** 2013-12-20

**Authors:** Diego Clemente, María Cristina Ortega, Carolina Melero-Jerez, Fernando de Castro

**Affiliations:** Grupo de Neurobiología del Desarrollo-GNDe, Hospital Nacional de ParapléjicosToledo, Spain

**Keywords:** multiple sclerosis, oligodendrogliogenesis, remyelination, molecular cues, astrocytes, microglia

## Abstract

Oligodendrocyte precursor cells (OPCs) originate in specific areas of the developing central nervous system (CNS). Once generated, they migrate towards their destinations where they differentiate into mature oligodendrocytes. In the adult, 5–8% of all cells in the CNS are OPCs, cells that retain the capacity to proliferate, migrate, and differentiate into oligodendrocytes. Indeed, these endogenous OPCs react to damage in demyelinating diseases, like multiple sclerosis (MS), representing a key element in spontaneous remyelination. In the present work, we review the specific interactions between OPCs and other glial cells (astrocytes, microglia) during CNS development and in the pathological scenario of MS. We focus on: (i) the role of astrocytes in maintaining the homeostasis and spatial distribution of different secreted cues that determine OPC proliferation, migration, and differentiation during CNS development; (ii) the role of microglia and astrocytes in the redistribution of iron, which is crucial for myelin synthesis during CNS development and for myelin repair in MS; (iii) how microglia secrete different molecules, e.g., growth factors, that favor the recruitment of OPCs in acute phases of MS lesions; and (iv) how astrocytes modify the extracellular matrix in MS lesions, affecting the ability of OPCs to attempt spontaneous remyelination. Together, these issues demonstrate how both astroglia and microglia influence OPCs in physiological and pathological situations, reinforcing the concept that both development and neural repair are complex and global phenomena. Understanding the molecular and cellular mechanisms that control OPC survival, proliferation, migration, and differentiation during development, as well as in the mature CNS, may open new opportunities in the search for reparative therapies in demyelinating diseases like MS.

## INTRODUCTION

Since the founding of neurosciences in the nineteenth century, neurons, the fast excitable cells of the nervous system, have captivated the attention of almost all the successive generations of researchers. The huge number of neurons, their fascinatingly precise interactions in the mature central nervous system (CNS), and the diversity of physiological responses and behaviors that they elicit, undoubtedly deserve such consideration. Nevertheless, the number of glial cells in the CNS is close to one order of magnitude higher than that of neurons. A recent estimate of the number of neurons in the mature human cerebral cortex was lowered to a few billion, being approximately sixfold more glial cells (Verkhratsky and Butt, 2007; Kandel et al., 2013; Ribeiro et al., 2013). In the last decade, there is even more evidence that all the major types of glial cells fulfill activities beyond their classically accepted functions. During normal adulthood and in response to pathological insults, mature oligodendrocytes are actively replaced by oligodendrocyte precursor cells (OPCs), a cell type that represents a highly relevant proportion of the total cells in the adult CNS (between 5% and 8%, depending on the structure and species studied; Dawson et al., 2000; Young et al., 2013). OPCs also receive synaptic inputs from neurons, although the functional role of this novel interaction has not been discovered yet (Frohlich et al., 2011; Passlick et al., 2013), and in response to neural activity, oligodendrocytes liberate exosomes that offer trophic support to myelinated axons (Fruhbeis et al., 2013).

In recent years, the amount of data indicating that both astrocytes and microglia influences oligodendrocyte biology has grown steadily. Far from their classic roles as physical scaffolds and their participation in nutrient diffusion throughout the CNS, astrocytes have now been shown to modulate neuronal activity and to respond to neurotransmitters in a clear and excitable manner (Araque and Navarrete, 2010). In addition, the role of microglia in response to CNS injury is now accepted to be much more complex than previously considered. Accordingly, it is becoming increasingly clear that microglia influence homeostasis by secreting diverse molecules that control different physiological processes, e.g., axon outgrowth, branching, inflammatory responses, etc. (Ransohoff and Perry, 2009). Hence, the purpose of the present work is to review the direct interactions between astrocytes/microglia and OPCs during normal CNS development and in pathological circumstances, focusing particularly on multiple sclerosis (MS).

Oligodendrocytes were originally identified by the great Spanish neuroscientist Pío (del Río-Hortega, 1921). They engage in complex interactions with the soma and the axon of neurons in the CNS, and their most singular characteristic is their ability to form myelin sheaths (Barres and Barde, 2000). Myelin represented a fundamental evolutionary advance given that it increases the efficiency and velocity of nerve impulse conduction (Zalc and Fields, 2000). Oligodendrocytes are distributed ubiquitously throughout the adult CNS, in both the white and gray matter (Miller, 1996), although they originate from OPCs in multiple but discrete foci along the neural tube during development (Miller, 2002; Qi et al., 2002; Spassky et al., 2002; de Castro et al., 2013). Once generated, OPCs proliferate in response to different mitogenic agents and, guided by a complex ensemble of signals, they disperse throughout the prospective gray and white matter to populate the developing CNS before they finally differentiate into mature oligodendrocytes (Rowitch, 2004; de Castro and Bribian, 2005; Furusho et al., 2011; de Castro et al., 2013). Two different populations of OPCs have been identified during development: (i) one type of OPC is characterized by the expression of *plp/dm-20* and these do not depend on PDGF-AA; (ii) the second and largest population of OPCs does depend on this growth factor and these cells express PDGFRα (Le Bras et al., 2005). OPCs compete for both space and trophic factors, and while ventrally generated OPCs predominate in the spinal cord, those generated dorsally are the most abundant in the telencephalic territories (Richardson et al., 2006; Tripathi et al., 2011; de Castro et al., 2013). Once at their final destination, oligodendroglial cells become mature in response to a combination of molecules (growth factors, hormones, neurotransmitters, extracellular matrix (ECM) proteins: Emery, 2010; Furusho et al., 2012), and they acquire their typical biochemical profile (myelin protein expression, including MBP, PLP, and MAG) and morphology, covering axons and forming myelin sheaths around them (de Castro and Zalc, 2013). During development, many important phases of oligodendrogliogenesis and myelination are controlled by the other two main glial cells, astrocytes, and microglia. These cells control the secretion and bioavailability of cues or other key factors (e.g., iron) that modulate the survival, proliferation, and migration of OPCs during the processes that lead to the production of a functional myelin sheath. In the two first epigraphs of this work, the role of astrocytes and microglia in these two important aspects will be extensively reviewed and discussed.

Oligodendrocytes may die in different pathological scenarios, such as primary demyelinating diseases (MS, adrenoleukodystrophies), traumatic and vascular accidents (spinal cord injury, skull trauma, cerebral infarct), neurodegenerative diseases, and schizophrenia (Edgar and Sibille, 2012; Goldman et al., 2012). The large number of OPCs that exist in the adult brain can orchestrate a reaction to such events, producing spontaneous remyelination and partial recovery of the oligodendrocyte lost (Piaton et al., 2009). The best studied demyelinating disease in humans is MS (the most frequent neurological disease in young adults), and the role of endogenous adult OPCs in the pathogenesis and recovery of MS demyelinating lesions is currently a very active field of research for modern neuroscientists and neurologists (Prineas and Parratt, 2012; Cui et al., 2013). The neuropathological events associated with MS include the infiltration of blood cells into the white matter, demyelination due to oligodendrocyte loss, and axon degeneration (Noseworthy et al., 2000; Compston and Coles, 2008; Henderson et al., 2009). Demyelinating lesions are classified as active, chronic-active, and chronic-inactive lesions, depending on their histopathological characteristics and their intrinsic capability for spontaneous remyelination, which leads to the formation of partially repaired shadow plaques (**Table 1**). Indeed, all three types of lesion plaques can be observed in the CNS of MS patients, independently of their clinical evolution and phenotype (Breij et al., 2008; Frischer et al., 2009; Bramow et al., 2010). This implies that during the development of a demyelinating lesion, not only inflammatory-infiltrated cells but also, CNS resident astrocytes and microglia, experiment changes in their activity and distribution that may affect OPCs through different mechanisms. In the third and fourth epigraphs of this review we will summarize what is currently known about the important effects of microglia and astroglia on OPCs in MS. We will focus on the secretion of molecules by the resident microglia that effectively recruits OPCs in order to replace dead oligodendrocytes at early (active) demyelinating lesions. We will also examine in detail how astrocytes modify the molecular environment as demyelinating lesions stabilize and become chronic, and their fundamental influence on OPCs that may limit their ability to invade the lesions or that arrests their differentiation into new myelin-forming oligodendrocytes. Finally, we discuss how all this knowledge could represent the basis to develop future therapeutic approaches that aim to partially or totally replace the dead oligodendrocytes.

**Table 1:** Summary of the histopathological characteristics of the multiple sclerosis lesion.

	Tissue region	Tissue characteristics	Myelin	Astrocytes	Microglia/macrophages	Other immune cells
Active	Plaque	Indistinct margin; edema; widespread axonal damage	Demyelination; OPC recruitment; occasional remyelination	Hypertrophic astrocytes; little astrocyte scarring	Strong microglial activation; myelin-laden macrophages	Vascular infiltration of small lymphocyte; plasma cells
Shadow	Plaque	Sharply demarcated; relative axonal preservation	≥60 % of remyelinated area; uniformly thin myelin sheaths	No apparent astrocyte activation	No microglial activation; absence of macrophages	Absent
Chronic-active	Plaque	Sharp edge; hypocellular; naked axons	Complete demyelination; absent remyelination; scarce OPCs	Scarring fibrous astrocytes	Few lipid-laden macrophages	Few infiltrating leukocytes
	Periplaque	Initial axonal damage	Demyelination; OPC recruitment; occasional remyelination	Hypertrophic astrocytes	Strong microglial activation; lipid-laden and myelin-laden macrophages	Perivascular cuff of infiltrated cells
Chronic-inactive (silent)	Plaque	Sharp edge; reduced number of demyelinated axons; thickened blood vessel wall	Complete demyelination; absent remyelination; scarce OPCs	Astrocytic glial scar	Little or no microglial activation; reduced number of macrophages	Occasional leukocytes

*OPC, oligodendrocyte precursor cell.*

## HOW ASTROCYTES INFLUENCE OLIGODENDROCYTE PRECURSOR CELLS DURING CNS DEVELOPMENT

During development, OPCs are generated in oligodendrogliogenic niches that form a patchwork pattern along the neural tube, from where they migrate to colonize the whole CNS (de Castro and Zalc, 2013). Along their complicated routes of migration, OPCs frequently come into contact with the surface of astrocytes and they use the astrocyte-derived matrix as a substrate for their movement (Schnadelbach and Fawcett, 2001). N-cadherins are relatively abundant on the surface of perinatal astrocytes and OPCs quickly adhere to them, spreading over their surface (Schnadelbach et al., 2000). However, establishing long-lasting firm contacts with astrocytes would anchor OPCs to these cells and prevent them from migrating further (Fok-Seang et al., 1995; Schnadelbach et al., 2000). In addition, there is abundant evidence that cues secreted by astrocytes influence the behavior of OPCs during development (Barres et al., 1993; Chernausek, 1993; Gard et al., 1995; Moore et al., 2011). Among these, PDGF-AA is one of the best studied as its secretion by astrocytes promotes the survival and proliferation of OPCs (Besnard et al., 1987; Raff et al., 1988; Richardson et al., 1988; Gard et al., 1995). Indeed, the effect of astrocyte-derived PDGF-AA in stimulating OPCs to either proliferate or differentiate into mature oligodendrocytes is influenced by the developmental stage of the progenitor cells (Raff et al., 1988), suggesting that in the optic nerve at least, PDGF-AA produced by astrocytes coordinates the timing of oligodendrogliogenesis (Durand and Raff, 2000).

Besides the cues that promote OPC proliferation, migration, and differentiation, there are inhibitory factors that also regulate these aspects of the oligodendrocyte biology, such as bone morphogenetic proteins (BMPs) and chemokines. BMPs are secreted by astrocytes (Chang et al., 2003; Miyagi et al., 2012) and they inhibit the differentiation of OPCs into myelin-producing oligodendrocytes (See et al., 2004; See and Grinspan, 2009). *In vitro* experiments have shown that BMP4 inhibits the expression of several myelin proteins during oligodendrocyte differentiation, such as PLP and MBP (See et al., 2004). Although there have been some *in vivo* studies into BMPs and oligodendrocyte development (Mekki-Dauriac et al., 2002; Miller et al., 2004), the regulation of OPC differentiation by astrocyte-produced BMPs has not yet been specifically examined. Chemokines are another well-known example of the molecular cues expressed by astrocytes that affects OPC behavior during development (Robinson et al., 1998; Tsai et al., 2002) and these molecules display a broad range of activities in different tissues (regulation of gene expression, cell adhesion, cell polarization, and chemotaxis), including the CNS (Baggiolini, 1998). During development, oligodendrocytes express at least two chemokine receptors, CXCR1 and CXCR2 (Nguyen and Stangel, 2001). The CXCR2 ligand, CXCL1 has been shown to promote the OPC proliferation *in vitro* (Robinson et al., 1998) and astrocytes transiently express high levels of CXCL1 during the development of the spinal cord (Tsai et al., 2002). Indeed, CXCL1-mediated signaling through CXCR2 on OPCs inhibits their migration, provoking intense proliferation by a PDGF-AA-driven mechanism (Tsai et al., 2002).

**FIGURE 1:**
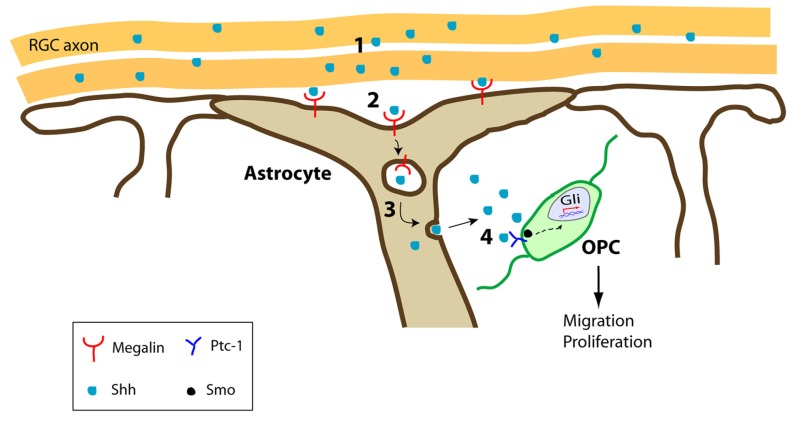
**Schematic representation of the optic nerve trafficking of Shh by megalin^+^-astrocytes.** (1) RGC axons secrete Shh during optic nerve development. (2) Shh is internalized by megalin^+^-astrocytes. (3) Shh is transcytosed across the astrocytes to be released. (4) Once released, Shh exerts its effects on Ptc-1^+^-expressing OPCs in the optic nerve, promoting their migration and proliferation. RGC, retinal ganglion cells.

We recently identified another way by which astrocytes control OPC behavior during development, by controlling the bioavailability of these cues. During the first stages of CNS development, a gradient of Shh must be established to correctly specify the ventral domains of the neural tube (Nery et al., 2001; Cohen, 2003; Bertrand and Dahmane, 2006; Dessaud et al., 2008). Moreover, at later developmental stages Shh participates in the proliferation and migration of OPCs during the colonization of the optic nerve (Gao and Miller, 2006; Merchán et al., 2007). Besides the canonical Shh receptor Patched-1 that is expressed by OPCs, the multiligand receptor megalin, a member of the low density lipoprotein receptor family (also known as gp330 or LRP-2), can also bind Shh (McCarthy et al., 2002; Ortega et al., 2012). Megalin is an important element in the molecular trafficking of different proteins within the cell and once internalized by megalin, such proteins can be redirected, released, or hydrolyzed depending on the cell type (Christensen and Birn, 2002; McCarthy and Argraves, 2003; Morales et al., 2006; Bento-Abreu et al., 2008). During optic nerve development, megalin is exclusively expressed by astrocytes, in a dynamic pattern that parallels the colonization of OPCs from the optic chiasm to the retina (Spassky et al., 2002; Ortega et al., 2012). When OPCs start invading the nerve, at E14.5 in the mouse, (Spassky et al., 2002), megalin is more profusely distributed in the caudal region of the nerve (close to the optic chiasm) than in the rostral part (close to the eye); this distribution is inverted at E16.5 (megalin accumulating more intensely near the eye) when the first OPCs are completing their journey to the retina. By the time OPC colonization of the optic nerve has been completed (E18.5), megalin is weakly and uniformly expressed all along the nerve (Ortega et al., 2012), a time when Shh no longer attracts migrating OPCs (Merchán et al., 2007). Moreover, *in vitro* experiments showed that blocking megalin hinders optic nerve OPC proliferation and migration, and megalin^+^-astrocytes can internalize Shh for its subsequent release at a suitable concentration (Ortega et al., 2012). Shh is homogeneously expressed by retinal ganglion cells and it is secreted by their axons at different developmental stages (Traiffort et al., 2001). Hence, megalin^+^-astrocytes can regulate the concentration of extracellular Shh available in order to create a functional gradient of this morphogen in the optic nerve, promoting its release to OPCs located far from the main source (**Figure 1**; Ortega et al., 2012).

In summary, most studies into oligodendrogliogenesis carried out to date have focused on the effects exerted by different cues, without taken into consideration the participation of other glial cells on the diverse facets of OPC biology. However, we have given some examples of how astrocytes regulate important aspects of oligodendrocyte behavior through the release of several molecular cues and by regulating their bioavailability. Hence, not only the cue but its cellular source appear to exert an active and crucial effect on OPCs during CNS development, which should be taken into account in future analyses.

## ASTROGLIA AND MICROGLIA REDISTRIBUTE IRON: A CRUCIAL PROCESS FOR MYELIN SYNTHESIS DURING CNS DEVELOPMENT AND MYELIN REPAIR IN MS

Oligodendrocytes are the main iron-containing cells in the CNS and iron is required as a co-factor for the enzymes involved in myelination (Todorich et al., 2009). During CNS development, iron^+^-oligodendrocytes are present in myelinogenic foci, reflecting the functional relationship between iron accumulation and myelin production (Connor and Menzies, 1996). Oligodendrocytes are not able to synthesize iron, so they must acquire it from the surrounding environment. Ferritin and transferrin are the most important proteins that deliver iron to the brain, the latter being crucial for iron to cross the blood–brain barrier (BBB) and enter the CNS (Leveugle et al., 1996). The brain is the only organ in which transferrin mRNA expression augments after birth, and this increase is directly related to oligodendrocyte maturation. However, the expression of transferrin receptors in oligodendrocytes decreases as the animal ages and it is not detected in adults. For this reason, it has been suggested that ferritin acts as an alternative iron transporter in adult oligodendrocytes and that ferritin becomes the dominant iron delivery protein in adults (Hulet et al., 1999).

Currently, oligodendrocytes appear to depend on two sources of iron delivery in the brain: (i) direct transport of exogenous ferritin/transferrin across the BBB (Fisher et al., 2007); and (ii) resident microglial cells as a source of ferritin (Zhang et al., 2006). During postnatal development, microglia represents the main iron source for oligodendrocytes. It has been shown that microglia accumulate iron just before myelination and that the decrease in microglia iron load is paralleled by the accumulation of iron in oligodendrocytes (Cheepsunthorn et al., 1998). These findings suggest that microglia first accumulate iron and that they then release it to the developing OPCs during myelination, when the iron requirement is maximum for their maturation (Todorich et al., 2009). In pathological scenarios, there is compelling evidence that the survival of oligodendrocytes is dependent on the release of ferritin by microglia (Zhang et al., 2006). Recently published *in vivo* data support the relevance of microglial cells as an iron source during oligodendrocyte proliferation (Schonberg et al., 2012). After ferritin microinjections into the spinal cord of adult rats it is rapidly internalized by microglia, and then subsequently released to promote the proliferation of NG2^+^-OPCs and their differentiation into mature oligodendrocytes. These data are consistent with the accumulation of ferritin^+^-NG2 cells close to an area of ferritin-expressing activated macrophages (Schonberg and McTigue, 2009). Together, these data indicate that in the adult CNS, microglial cells might act as a source of iron for NG2^+^-cells, thereby contributing to tissue repair by stimulating proliferation and the formation of new myelin-producing oligodendrocytes.

On the other hand, most of the iron found in the human brain parenchyma is stored as non-heme iron in oligodendrocytes and it is essential for normal brain metabolism (Rouault and Cooperman, 2006). Ferrous iron ions (a liberated form of iron) must be strictly regulated since they may generate toxic reactive oxygen species (ROS), with oligodendrocytes being especially vulnerable to such injury (Connor and Menzies, 1996; Todorich et al., 2009). Indeed, abnormally high levels of iron have been detected in both the gray and white matter of MS patients (LeVine, 1997). Since iron can facilitate the polarization of microglia, adopting a pro-inflammatory phenotype, and act as a catalyst for ROS production, the liberation of the iron accumulated in the brain may participate in demyelinating and neurodegenerative processes (Williams et al., 2012; Hametner et al., 2013). The distribution of iron differs in MS lesions and normal-appearing white matter (NAWM), and the oligodendrocytes located at the MS lesion edge have a reduced iron load compared to those in the NAWM. By contrast, microglia iron content increases in an opposite manner, i.e., it is higher close to the periplaque than in the NAWM (Hametner et al., 2013). This indicates a clear relationship between the exacerbation of the pro-inflammatory environment and the accumulation of iron by microglia (or macrophages) in MS tissue, as shown previously through several *in vitro* and *in vivo* approaches (Schonberg and McTigue, 2009; Rathore et al., 2012). The limit of this situation lies at the innermost portion of the active lesion where extracellular iron deposits are dense and iron-containing oligodendrocytes are shown to be damaged and disappear (Hametner et al., 2013). Together, iron accumulation by microglia in the periplaque and NAWM could be understood as a buffering mechanism to limit the vulnerability of oligodendrocytes to oxidative damage. However, the suggestion of enhanced pro-inflammatory cytokine release by iron-loaded cultured microglia (Zhang et al., 2005) highlights the need to understand the relationship between iron status and oligodendrocyte damage in *in vivo* models of demyelination. In summary, the role of iron in MS is a source of controversy: while the presence of iron in lesions may increase OPC proliferation and differentiation, it might also have negative effects by promoting pro-inflammatory cytokine release by microglia and the presence of a highly oxidative environment that compromises oligodendrocyte survival. Hence, further experiments are needed to shed more light on how iron influences the relationship between microglia and OPCs in de- and remyelinating scenarios.

A further twist in the complex relationship between glial cells that controls the accumulation of iron arises when we consider the ideal position of astrocytes to take up circulating iron and distribute it to other cells via the iron exporter, ferroportin (Jeong and David, 2003). Indeed, the induction of toxin-driven demyelination in an astrocyte-specific ferroportin knockout mouse produces a clear decrease in the rate of remyelination due to reduced OPC proliferation (Schulz et al., 2012). It was suggested that this may reflect a direct effect of iron on OPCs (direct iron deficiency in this cell type) or an indirect effect of iron deprivation in microglial cells. Iron deprivation would affect the activation state of microglia, reducing the secretion of pro-inflammatory cytokines and reciprocally down-modulating the production by astrocytes of growth factors involved in OPC proliferation (IGF-1 and FGF-2). Hence, there is clearly a need for more detailed research in this area in order to understand the true role of iron homeostasis in the biology of all glial cell types, and its consequences in diverse pathological scenarios, including MS.

## MICROGLIA INDUCE THE PROLIFERATION AND RECRUITMENT OF OPCs IN EARLY MS LESIONS

### MICROGLIAL PHENOTYPES AS REGULATORS OF THE INFLAMMATORY ENVIRONMENT IN MS

**FIGURE 2:**
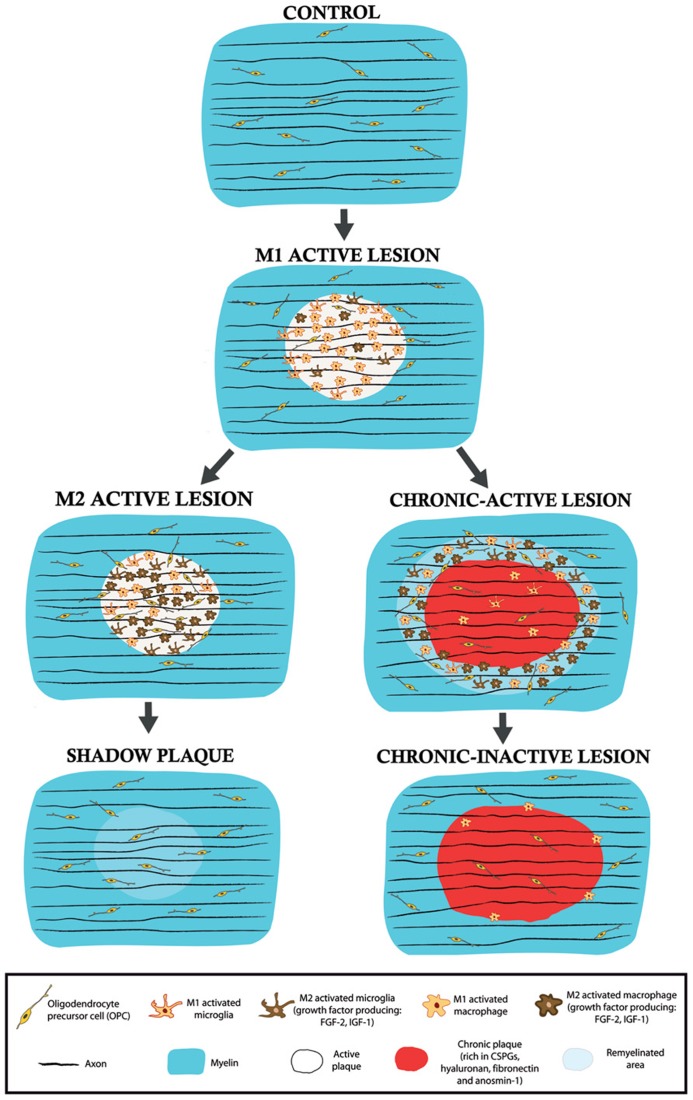
**Schematic drawings of the different types of lesions in the MS white matter.** M1 active lesions are characterized by the presence of microglia/macrophage in a pro-inflammatory activity state. In these lesions, remyelination does not occur and demyelination advances. According to the results observed in a murine model of focal demyelination (Miron et al., 2013), an active lesion may derive into a M2 predominant activity state and thus, microglia/macrophages would change their phenotype and begin to release pro-regenerative molecules, such as the growth factors IGF-1 or FGF-2. If this activity persists, partial remyelination may exist in the so-called shadow plaque. By contrast, if the myelin-destructing environment takes hold, an active lesion may evolve into a chronic-active lesion in which remyelination can mainly occur in the periplaque (with M2 microglia/macrophage growth factor release) whereas the plaque is devoid of remyelinating profiles due, in part, to the blockade of OPC invasion and differentiation provoked by unusual astroglial ECM deposition. In chronic-inactive lesions, inflammatory and demyelinating activity ceases in both areas.

It is becoming more established that the regeneration observed in MS lesions, i.e., remyelination, may be a consequence of changes in the local environmental equilibrium between pro-inflammatory/pro-myelin-damage and anti-inflammatory/pro-regenerative molecules/cells (Rawji and Yong, 2013). Fundamental components of this functional switch are the cells of the innate immune system, mainly CNS-resident microglia and their peripherally derived counterparts, macrophages. Microglia are important elements in the innate immune system that are located within the CNS, and that influence the adaptive immune response and the biological activities of other resident CNS cells (Ransohoff and Perry, 2009). Classically, microglia (and macrophages) have been exclusively considered in a negative light in MS, based on their capacity to secrete toxic molecules and present antigens to cytotoxic lymphocytes (Banati et al., 1993; Cash et al., 1993). In recent years, it has been shown that activated microglia probably facilitate remyelination in MS and for example, phagocytosis of myelin debris is necessary for complete remyelination (Li et al., 2005; Kotter et al., 2006; Setzu et al., 2006). In addition, regenerative microglia/macrophages enhance growth and neurotrophic factor production (Kotter et al., 2005; Clemente et al., 2011). Although controversial (Block et al., 2007; Hanisch and Kettenmann, 2007), recent data suggest that microglia can fulfill both roles, showing physiologically different phenotypes as regulators of degenerative or regenerative processes (Miron et al., 2013). This clearly indicates the existence of at least two activated states of microglia and macrophages: (i) the classical activated state or M1, that is associated with enhanced antigen presentation and the secretion of pro-inflammatory cytokines, as well as reactive oxygen and nitrogen species (Edwards et al., 2006); and, (ii) the alternative/de-activating M2 polarized phenotype, which secretes anti-inflammatory cytokines and growth factors, that participates in tissue regeneration by limiting inflammation, and that also induces OPC recruitment, proliferation, and differentiation (Edwards et al., 2006; Miron et al., 2013). Significantly, both fetal and adult isolated human microglia and macrophages have recently been shown to polarize to a M1/M2 phenotype depending on the local environment they are cultured in (Durafourt et al., 2013). It is important to note that the molecules that M1 and M2 cells secrete reciprocally control their activated states in the CNS, in what represents a clear example of a homeostatic equilibrium aimed at controlling tissue damage (Das et al., 2001; Jang et al., 2013). Moreover, it has been demonstrated that both activated resident microglia and infiltrated activated macrophages can switch dynamically from an M1 to a M2 phenotype in a demyelinating environment *in vivo* and *in vitro* (Miron et al., 2013). In this sense, growth factor secretion is suppressed in a pro-inflammatory (M1) environment but it is encouraged in a M2 anti-inflammatory scenario, with fine reciprocal control (Wynes and Riches, 2003). Therefore, a disturbed balance among interacting growth factors that regulate OPC proliferation, migration, differentiation, and hence, the onset of myelin formation, may contribute to the limited remyelination observed in MS lesions (**Figure 2**).

### FACTORS SECRETED BY MICROGLIA AND THEIR INFLUENCE ON ENDOGENOUS REMYELINATION

IGF-1 is one of the growth factors that has been studied most extensively in the CNS, being produced putatively by M2 microglia/macrophages to promote myelin repair. It enhances the survival of oligodendrocytes, promotes oligodendrocyte development, and stimulates the synthesis of myelin (McMorris et al., 1986; Barres et al., 1992; Roth et al., 1995; Mason et al., 2000). It is likely that IGF-I acts through IGF-receptor 1 (IGFR1) on the surface of cells of the oligodendrocyte lineage, since OPC proliferation is diminished and apoptosis is enhanced in an IGFR1 conditional knockout mice for this lineage, resulting in fewer mature oligodendrocytes (Zeger et al., 2007). IGF-1 not only protects myelin from different types of insults (Cao et al., 2003; Wood et al., 2007) but also, it is known to promote oligodendrocyte proliferation and differentiation from multipotent precursor cells (Mason et al., 2000; Hsieh and Papaconstantinou, 2004). After cuprizone-induced demyelination, the lack of IGF-1 in an IL-1β knockout mice parallels the delay in OPC differentiation into mature oligodendrocytes (Mason et al., 2001), whereas its overexpression promotes remyelination by enhancing the survival of myelinating oligodendrocytes (Mason et al., 2000). In several toxin-induced models of demyelination, IGF-1 is up-regulated in microglial cells during the stages of myelin loss, peaking at the end of the demyelination period when the first remyelinating profiles appear (Hinks and Franklin, 1999; Fushimi and Shirabe, 2004; Gudi et al., 2011; Voss et al., 2012). By contrast, in MS tissue IGF-1 was first detected in hypertrophic astrocytes but not in macrophages or microglia within active lesions, and its receptor IGFR1 was observed in macrophages and in a subpopulation of astrocytes, yet not in oligodendrocytes within plaques (Gveric et al., 1999). Subsequently, both IGF-1 and IGFR1 were found in oligodendrocytes at the edge of chronic lesions, as well as in the NAWM, albeit expressed much more strongly in the former (Wilczak et al., 2008). Therefore, the data from animal models and human tissue differ, which may reflect the simplicity of a weakly stimulated immune system model (i.e., toxin-induced), and the complexity of different cells and processes detected in the human MS tissue.

Among the growth factors studied to date, FGF-2 is considered to be one of the most controversial in terms of myelin repair. Undoubtedly, FGF-2 is one of the main mitogens for OPCs and it participates as a motogenic/chemokinetic and chemotropic factor for OPC migration during development (Bogler et al., 1990; McKinnon et al., 1990; Bribián et al., 2006), a property that it retains in adulthood (Clemente et al., 2011). This latter activity is mediated by its binding to FGFR1, which is expressed by both embryonic and adult OPCs (Bansal et al., 1996; Bribián et al., 2006; Clemente et al., 2011). However, other effects of FGF-2 on oligodendroglial cells have been questioned, i.e., differentiation. The first reports indicated that FGF-2 arrests OPC differentiation *in vitro* in normal or pseudo-pathological conditions (Goddard et al., 1999, 2001). However, following the deletion of both *fgfr1/fgfr2* in the oligodendrocyte lineage, it appears that FGF signaling is required for correct myelination without affecting oligodendrocyte proliferation (Furusho et al., 2012). Thus, FGF-2 may be involved specifically in oligodendrocyte responses during demyelination and remyelination. However, this growth factor has controversial effects during regenerative processes in different demyelinating animal models with no or low interference of immune response (Armstrong et al., 2002; Butt and Dinsdale, 2005; Tobin et al., 2011). By contrast, it seems that in the immune-associated demyelinating model EAE, FGF-2 controls the immune system response and cell infiltration, which provokes a protective oligodendrocyte effect (Ruffini et al., 2001; Rottlaender et al., 2011). Hence, FGF-2 would be another putative candidate to fine tune the regulation of the M1/M2 phenotype in complex autoimmune demyelinating diseases, like MS.

The source of FGF-2 varies in function of the demyelinating models explored. Enhanced expression of this growth factor was detected in microglial cells within spinal cord lesions due to lysolecithin-induced demyelination (Hinks and Franklin, 1999), and in the corpus callosum of cuprizone-induced demyelinating lesions at the time when remyelination starts (Gudi et al., 2011). Conversely, FGF-2 was found in astrocytes after a hepatitis virus-induced demyelination in mice (Messersmith et al., 2000), whereas in EAE, FGF-2 was mainly described in microglia and macrophages associated with demyelinated areas (Liu et al., 1998). We recently shed some light on the controversial adscription of the cell source of this growth factor by describing the pattern of FGF-2 expression in different MS lesions (Clemente et al., 2011). Although almost absent in control subjects, FGF-2 was detected in a subpopulation of CD68^+^HLA-DR^+^-macrophages or microglia within active lesions and in the periplaque of chronic-active lesions, both areas where remyelination occurs spontaneously (Frohman et al., 2006). Interestingly, FGF-2 almost disappeared from partially remyelinated shadow plaques, with no differences between the NAWM and the remyelinated area in terms of the distribution and shape of the scarce FGF-2-containing microglial cells. It is particularly interesting that those areas where remyelination is compromised (the core of chronic lesions) are devoid of FGF-2-producing cells (Clemente et al., 2011). Therefore, a clear parallel exists between the spontaneous remyelination associated with a specific lesion and the presence of FGF in macrophage/microglial cells. Identifying the elements controlling the M1/M2 characteristics of the FGF-2 expressing macrophages/microglia, as well as those involved in establishing their resident-microglial or infiltrating-macrophage origin, will require further analysis.

Interestingly, we revealed a gradient in the density of FGFR1 expressing PDGFRα-OPCs in human tissue for the first time, from more numerous within areas where FGF-2-expressing macrophages were present to rare in the adjacent NAWM (Clemente et al., 2011). This observation, together with the aforementioned conservation of the chemoattractant capacity of FGF-2 on adult OPCs (Clemente et al., 2011), represents one of the first examples in human of the interesting relationship between putative M2 regenerative macrophages/microglia and reparative OPCs, exclusively in those areas where remyelination may occur. Indeed, FGFR1 up-regulation on OPCs may be due to FGF-2 itself (Bansal et al., 1996), or it may be the result of the existence of two different subpopulations of FGFR1^+^- and FGFR1^-^-OPCs with specific proliferative, migratory and/or differentiating responses to this growth factor. All these considerations should be carefully taken into account in order to design new growth factor strategies to treat a complex disease like MS.

## ASTROCYTES MODIFY THE EXTRACELLULAR MATRIX IN ADVANCED MS LESIONS, AFFECTING SPONTANEOUS REMYELINATION

As mentioned above, spontaneous remyelination associated with active MS lesions is possible under specific circumstances (Frohman et al., 2006; Patrikios et al., 2006; Patani et al., 2007). However, in chronic MS lesions remyelination is absent or mainly detected in the plaque border (Prineas and Connell, 1979; Barkhof et al., 2003; Bramow et al., 2010), probably due to the blockade of OPC migration into this area or to the arrest of their differentiation once they invade it (Franklin and Ffrench-Constant, 2008; Kuhlmann et al., 2008; Clemente et al., 2011). This implies complex changes in the lesion environment, e.g., in the ECM, mainly due to alterations in the astrocyte secretion profile (Sobel and Mitchell, 1989; Gutowski et al., 1999; Back et al., 2005; Van Horssen et al., 2005, 2006; Satoh et al., 2009).

The ECM is the ground substrate found in the interstitial spaces of all organs that provides support to its cells. In the CNS, the ECM is secreted by different cell types, including astrocytes, and it has traditionally been considered to play a predominantly structural role. However, recently new characteristics of the ECM have emerged and it has been shown that the composition of the ECM is strongly modified in MS, affecting both parenchymal and basement membrane components (Van Horssen et al., 2005, 2006). Several studies have considered how the astrocyte-secreted ECM influences the behavior of OPCs in MS (Maier et al., 2005; Clemente et al., 2011). Changes in the composition of the ECM due to astrocyte secretion during the evolution of a given lesion may convert a permissive environment for myelin repair into an inhibitory area, depending on the protein composition and the receptors expressed by the OPCs (de Castro et al., 2013). Nonetheless, in many cases, the role of different protein components of the ECM has been studied separately in both glial cell types (the responder OPC or the producer, mainly astrocytes), or only partially in the different aspects of oligodendroglial development (proliferation, migration, differentiation), without analyzing the consequences or the relationship between both cell types.

The first *in vitro* evidence of such a tight relationship came more than 10 years ago (Schnadelbach et al., 2000; Maier et al., 2005). Subsequently, the progressive death of mouse oligodendrocytes in culture was shown to be suppressed by laminin-containing astrocytes through a mechanism that was dependent on the integrin receptor composition of the OPC cell membrane (Corley et al., 2001). Oligodendrocyte loss and microglial activation without strong participation of the immune system occurs in the cuprizone demyelination model (Matsushima and Morell, 2001), making it a good model to characterize the astroglial response during demyelination/remyelination. In this model, astrocytes exhibit stronger expression of fibronectin and chondroitin sulfate proteoglycans (CSPGs) with a dense elaboration of GFAP- and vimentin-containing processes (Hibbits et al., 2012). Similar changes in ECM composition have been obtained in the Theiler’s virus murine model of encephalomyelitis (TMEV), involving an accumulation of CSPGs (decorin and neurocan), glycoproteins (laminin, entactin, tenascin-C, and fibronectin) and collagens (I and IV), paralleling the spatial and temporal development of astrogliosis within the demyelinated areas of the spinal cord (Haist et al., 2012).

### CHONDROITIN SULFATE PROTEOGLYCANS

CSPGs are a large family of ECM macromolecules, composed of a central core protein to which varying numbers of glycosaminoglycan side chains are attached. These proteins are expressed ubiquitously throughout the CNS and they fulfill critical roles in CNS development, plasticity, and post-injury responses (Laabs et al., 2007), as well as inhibiting the outgrowth of OPC processes, OPC differentiation, and adhesion (Siebert and Osterhout, 2011). Surprisingly, there is little information regarding the source of these macromolecules in both animal models and MS tissue. In MS lesions, there are fewer CSPGs in active lesions due to the action of myelin-phagocytosing foamy macrophages (Sobel and Ahmed, 2001). By contrast, in the lysolecithin-induced demyelination model CSPGs are over expressed in microglial cells in the core, and in astrocytes at the border of the demyelinated areas, indicating that CSPGs can probably be deposited by different cell types (Lau et al., 2012). Hyaluronan is a specialized glycosaminoglycan that binds CSPGs and this molecule is a paradigmatic example of the changes in ECM content during the evolution of a demyelinating area in EAE. Hyaluronan is secreted in a low molecular weight form by lymphocytes and macrophages in early lesions, while it is deposited in a higher molecular weight form by activated astrocytes in aged lesions. In MS tissue, the high molecular weight form is mainly detected at the core of chronic lesions, which is not as permissive to remyelination as the core (Back et al., 2005). It was suggested that in chronic demyelinated lesions, astrocyte-derived hyaluronan may inhibit OPC maturation, thereby blocking remyelination (Back et al., 2005). In this model the mechanism controlling failed remyelination in MS involves hyaluronan degradation by hyaluronidases, which generate hyaluronan oligomers that block OPC maturation and remyelination through TLR2-MyD88 signaling (Sloane et al., 2010).

In recent years, further insight into the influence of CSPGs and CSPG-related molecules on remyelination has opened exciting perspectives, given that the specific branching of CSPGs also appears to control OPC differentiation towards myelin-producing cells. Glycans, the complex molecules that are made up of sugar chains of varying lengths, are gaining importance in neural cell interactions during demyelination. In the cuprizone model, *N*-acetylglucosaminyltransferase-IX (GnT-IX) and therefore, the presence of branched *O*-mannosyl glycans on astrocytes, impeded OPC differentiation and therefore, reduced the remyelination rate (Kanekiyo et al., 2013).

### FIBRONECTIN

Another fundamental component of the ECM is fibronectin (Stoffels et al., 2013b), which has been implicated in OPC migration *in vitro* and *in vivo* (Sheppard et al., 1991; Milner et al., 1996), possibly preventing premature fetal myelin formation rather than participating in myelin formation *per se* (Buttery and Ffrench-Constant, 1999). In recent years, growing evidence indicate that fibronectin may inhibit the outgrowth of oligodendrocyte processes and myelin sheath formation in the white matter (Siskova et al., 2009), and that aggregated fibronectin inhibits myelin formation in different experimental paradigms (Stoffels et al., 2013a,b). As fibronectin is not expressed in the CNS of healthy adult humans, its presence in lesions may interfere with OPC differentiation, thereby impairing remyelination. In MS, fibronectin rapidly accumulates as an acute response to demyelination, although it is broken down during remyelination. However, in chronic lesions, astrocyte-released aggregated fibronectin persists (Stoffels et al., 2013a). Interestingly, cultured astrocytes produce fibronectin in a diffuse pattern in standard conditions, whereas it is deposited in fibril-like structures in inflammatory conditions. This explains why fibronectin transiently appears in a soluble state in demyelinated areas of the lysolecithin infusion model, while fills the infiltrated area in an aggregated form in the chronic relapsing EAE MS model (Stoffels et al., 2013a). Like many other ECM proteins, fibronectin binds integrins, an important family of receptors present in many cell types, including oligodendrocytes, that fulfill important roles in CNS myelination during development and in adulthood (for reviews on this topic see O’Meara et al., 2011; Ahrendsen and Macklin, 2013).

### ANOSMIN-1

We have studied the role of anosmin-1 in OPC development, another component of the ECM that is defective in the X-linked form of Kallmann syndrome (Soussi-Yanicostas et al., 2002; Dode and Hardelin, 2009). During development, anosmin-1 is expressed by both astrocytes and oligodendrocytes of diverse nerves and tracts in the CNS (Clemente et al., 2008; Gianola et al., 2009), as well as in axonal bundles at the time when OPCs colonize them (Bribián et al., 2006, 2008). During CNS development, anosmin-1 partially blocks the motogenic effect of FGF-2 on OPCs, a role that is conserved throughout lifespan. These effects were demonstrated to be FGFR1-dependent (Bribián et al., 2006; Clemente et al., 2011). Anosmin-1 participates in OPC adhesion in a manner that is independent of FGF-2 (Bribián et al., 2008), its different structural domains interacting with several other ECM proteins, including anosmin-1 itself (Bribián et al., 2008; Murcia-Belmonte et al., 2010). Although the cellular source of anosmin-1 has proven difficult to define in human tissue (Lutz et al., 1994; Duke et al., 1995), it may be synthesized by astrocytes, as occurs during the development of the cerebellum (Gianola et al., 2009). In MS, anosmin-1 is absent from active lesions or shadow plaques (i.e., areas where remyelination occurs), whereas it is found in the core of chronic-active and chronic-inactive plaques (areas where remyelination is compromised: Clemente et al., 2008). The presence of anosmin-1 in chronic MS plaques may impede OPC colonization rather than inhibit their differentiation. The effects of anosmin-1 on adult human OPCs in MS may be governed by FGFR1, since this receptor is up-regulated in this cell type in the periplaque of chronic lesions, while is not present within chronic lesions (Clemente et al., 2011). Indeed, this may be one of the reasons OPC colonization of this specific region is obstructed in humans, thereby blocking axonal remyelination.

In the past few years, the influence of ECM content on the shape of oligodendrocytes has become evident and it was shown that the surface area of this cell type critically depends on actomyosin contractility, which is regulated by the physical properties of the supporting matrix (Kippert et al., 2009). It was also demonstrated that the presence of astrocyte-produced ECM proteins with non-permissive growth properties in the CNS blocks oligodendrocyte surface spreading, which is accompanied by changes in the rate of endocytosis (Kippert et al., 2009). One implication of these findings would be that changes in the rigidity of the scarred MS lesion, an effect of the proteins secreted by reactive astrocytes, may shift the balance of the intracellular and extracellular forces, thereby inhibiting oligodendrocyte differentiation. Thus, the presence or persistence of an ECM protein in the core of chronic MS lesions could cause an increase in the rigidity of the demyelinated area compared to the surrounded periplaque, producing changes in the cellular forces that might drive the inhibition of remyelination. Another possibility that has been unexplored to date, is that, although these extracellular cues might not alter the physical properties of the demyelinated area, they would change the activity of signaling molecules that regulate intracellular forces (specifically those related to RhoA), thereby also inhibiting remyelination (Bauer and Ffrench-Constant, 2009; Baer et al., 2009). Anosmin-1 would be a firm candidate to mediate this phenomenon since it induces cytoskeletal rearrangements through FGFR1-dependent mechanisms involving Cdc42/Rac1 activation, two members of the Rho family of small GTPases (González-Martínez et al., 2004). However, further experiments are needed to establish whether anosmin-1 participates in controlling actomyosin contractility and thus, oligodendrocyte cell shape and differentiation.

## CONCLUDING REMARKS

**FIGURE 3:**
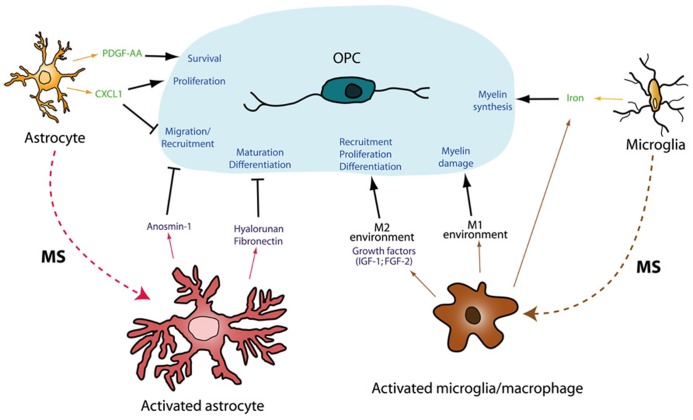
**Schematic representation of glia-glia interactions during development and pathology.** Myelination is the result of a well orchestrated variety of processes affecting OPC biology (several of them are summarized on the blue shadow). During development, astrocytes and microglia release several molecules (in green) which controls diverse aspects of the oligodendrogliogenesis. Both astrocytes and microglia are able to turn into an activated state in pathological conditions, such as MS. These activated cells can produce molecular factors (purple), which exert their effects on adult OPCs, enhancing (arrows) or impairing (bars) all the processes related to myelin synthesis and repair.

One of the most fundamental questions in neuroscience is whether it is possible to resolve pathological questions by recapitulating data obtained during the analysis of the CNS development. In this review we describe how microglia and astrocytes establish different interactions with OPCs, both in the normal CNS and in pathological conditions (a summary is described in **Figure 3**). Different astrocyte-secreted cues during development (semaphorins, Shh, FGF-2) influence the behavior of OPCs, either directly or indirectly, driving the response to important signals and/or helping to generate their gradients *in vivo*. Moreover, controlling iron homeostasis by microglia and astrocytes is not only essential for OPCs during normal myelination (Todorich et al., 2009) but also, in pathological circumstances (Williams et al., 2012). The overproduction of developmental growth factors by microglia/macrophages in areas where remyelination succeeds (Clemente et al., 2011) seems to be crucial for myelin sheath repair. This microglia–OPC interaction may represent a promising way to enhance the endogenous capacity to replace dead oligodendrocytes. Conversely, *de novo* deposition of different adhesion molecules by astrocytes in regions where remyelination does not spontaneously occur (Sobel and Mitchell, 1989; Back et al., 2005; Clemente et al., 2011; Stoffels et al., 2013a) could be interpreted as returning to a general developmental program to protect neural cells from extensive damage with non-desirable consequence for OPCs: the arrest of their migration and differentiation that impairs myelin repair. Hence, further studies about OPC biology should address: (i) the effects of the different molecules important for oligodendrogliogenesis during development (ECM proteins, secreted growth factors, secreted chemotropic molecules) on the biology of adult OPCs; (ii) the potential benefits of microglia as biological pumps for the *in situ* release of molecules that could reinforce myelin repair; (iii) the consequences of functionally silencing the signaling pathways in astrocytes that represent an obstacle for neuroprotection and remyelination in chronic MS lesions. In this sense, current MS therapies hinge on the supposition that controlling the inflammatory response will not only limit cell damage by inactivating different components of the innate or adaptive immune system, but that it will also have positive consequences on neuro-repair. However, the latter hypothesis has yet to be studied in MS tissue. Thus, it would be important to assess the specific effects of immunoregulatory molecules on the behavior of adult human OPCs and moreover, the effect of these current MS treatments on microglia and astrocytes, now clearly established as crucial partners in all the biological processes involved in OPC development: proliferation, migration, differentiation, and survival.

## Conflict of Interest Statement

The authors declare that the research was conducted in the absence of any commercial or financial relationships that could be construed as a potential conflict of interest.

## AUTHOR CONTRIBUTIONS

Diego Clemente wrote the manuscript, designed its content and prepared the figures; María Cristina Ortega wrote the manuscript and prepared the figures; Carolina Melero-Jerez wrote the manuscript; Fernando de Castro wrote the manuscript, designed its content and prepared the figures.
